# Ultrastructure of the Eggs, Larvae, and Pupae of *Hyphantria cunea* (Lepidoptera: Erebidae)

**DOI:** 10.3390/insects16020157

**Published:** 2025-02-03

**Authors:** Han Xue, Xinqian Liang, Qi Xie, Liu’er Yang, Mengcheng Wan, Cuiqing Gao

**Affiliations:** Co-Innovation Center for Sustainable Forestry in Southern China, College of Forestry and Grassland, Nanjing Forestry University, Nanjing 210037, China; xuehann23@163.com (H.X.);

**Keywords:** micropylar region, antennae, mouthpart, head chaetotaxy, prolegs, verrucae, ultramorphology

## Abstract

*Hyphantria cunea* is a widespread pest that causes significant issues in forestry and agriculture, with a far-reaching global impact. However, ultramorphological studies of *H. cunea*, especially in terms of ultramorphological changes, are far from in-depth. Therefore, we investigated the ultrastructure of its eggs, larvae, and pupae using scanning electron microscopy, focusing on ultrastructural changes in larvae across different instars. The eggs have rough surfaces, and the micropylar region consists of rosette cells. The larval head chaetotaxy is symmetrically arranged, and there are three types of sensilla on the antennae and mouthparts. The abdominal and anal prolegs feature a uniordinal heteroideus midband of crochets. The pupal abdomen has ten segments; in females, the genital slit (Fgs) is located at the anterior edge of abdominal segment VIII, while in males, the genital slit (Mgs) is situated in the middle of abdominal segment IX. First-instar larvae exhibit only primary setae, and secondary setae emerge in the second instar. As the larvae mature, the head, thoracic legs, and abdominal and anal prolegs all become larger overall. The surface of the verrucae is initially smooth, with microtrichia appearing from the fourth instar onwards, and the setae on the verrucae gradually increase in size. These findings provide additional features for larval species identification and contribute to the morphological study of *H. cunea*.

## Simple Summary

To enhance our understanding of the external morphology of *Hyphantria cunea*, we employed scanning electron microscopy to examine its eggs, larvae, and pupae. The results indicate that the eggs have rough surfaces, and the micropylar region is composed of rosette cells (Mrc). The head chaetotaxy of the larva is symmetrically distributed, with three types of sensilla on the antennae and mouthparts. The abdominal and anal prolegs possess a uniordinal heteroideus midband of crochets. The pupal abdomen consists of ten segments, with female and male pupae differing significantly in terms of the position and length of their genital slits. We also discussed the ultrastructural changes in *H. cunea* larvae across different instars. This information builds upon previous studies of *H. cunea* morphology, laying a solid foundation for the identification of larval species and providing a strong basis for the subsequent study of *H. cunea*.

## 1. Introduction

*Hyphantria cunea* is a significant quarantine pest globally, belonging to the Arctiinae, Erebidae, and Lepidoptera families. *H. cunea* is Holarctic and widely distributed in several countries. It was first identified in 1773 and was later discovered in North America and Europe in 1899 and 1940, respectively. By 1945, *H. cunea* had been introduced in Japan, where it caused considerable damage to fruit trees and other plants and was subsequently added to quarantine lists [1]. The pest spread to South Korea in 1958 and was first reported in Liaoning Province, China, in 1979. Thus far, *H. cunea* has invaded twenty European countries, including Austria, Italy, Germany, Russia, Romania, Hungary, Ukraine, and France [2]. *H. cunea* is a typical, rapidly spreading polyphagous pest that is capable of harming or even killing over 630 plants [3]. The larvae primarily inflict damage while feeding: first- and second-instar larvae only consume mesophyll, avoiding the veins and leaving leaves translucent; third-instar larvae create notches in leaves; fourth-instar larvae cluster within webbing; and fifth-instar larvae break through this webbing. The oldest larvae consume significantly more food prior to pupation. In severe cases, they can completely defoliate plants and transfer to another, causing widespread damage [4]. This pest poses a serious threat to ecosystems and economic development.

To date, research on *H. cunea* in both domestical and international contexts has primarily focused on its life history [5], morphological characteristics [6], control methods [7,8,9], invasive distribution and range [10,11], biological traits [12,13], population genetic differences [14], plant resistance [15,16], and genetic engineering [17]. Studies on the morphological characteristics of *H. cunea* mainly address the species identification of adults, larvae, pupae, and eggs [18], as well as the ultrastructure of larval and adult antennae, corpus allatum, and other sensory structures [19,20]. However, there is a lack of detailed reports on the ultrastructural changes in larvae across different instars. Similarly, descriptions of eggs and pupae are scarce.

This study investigates the eggs, larvae, and pupae of *H. cunea* using scanning electron microscopy, detailing the morphological variations from the first- to sixth-instar stages. This study will provide a theoretical basis for the ultramorphology of *H. cunea* and lay the groundwork for further research into their identification, physiological and biochemical functions, sensory mechanisms, feeding behaviors, and control methods.

## 2. Materials and Methods

### 2.1. Specimen Collection

Specimens were obtained from captive populations belonging to the Chinese Academy of Forestry (CAF) and wild populations in Shanghe County, Jinan City, Shandong Province, China. Some eggs were randomly selected for observation and preserved in 75% ethyl alcohol, and the remainder were reared in clean plastic boxes within an artificial climate incubator (RXZ-380B, Ningbo Southeast Instrument Co., Ltd., Ningbo, China) at a temperature of 25 °C ± 1 °C, with a relative humidity of 60% ± 10% and a photoperiod of L/D = 16h/8h. After hatching, the larvae were fed with fresh mulberry leaves. Healthy larvae of each instar were randomly collected and also preserved in 75% ethyl alcohol. After the larvae pupated, all pupae were collected and deformed or undersized pupae were discarded. Selected healthy pupae were sterilized via immersion in a 0.5% sodium hypochlorite solution, washed with distilled water, and dried in an oven at 70 °C.

### 2.2. Scanning Electron Microscopy (SEM)

The preserved eggs and larvae were processed in an ultrasonic cleaner for 10 min, and any surface dirt was removed with distilled water. They were then dehydrated through a graded ethanol series (30%, 50%, 70%, 80%, 90%, 95%, and 100%) for 20 min at each concentration. All specimens were then sputter-coated with gold and examined under a FEI Quanta 200 (FEI Company, Hillsboro, OR, USA, electronic imaging, SE) scanning electron microscope (hv, 10.00 kV; wd, 10.00 mm; spot 4.0).

### 2.3. Data Acquisition

In this study, 73 specimens at different stages were selected (eggs: 8; first instar: 12; second instar: 10; third instar: 10; fourth instar: 10; fifth instar: 8; sixth instar: 8; pupa: 7). The description of the specific details of each structure comes from the sixth-instar larvae. Measurements were taken from 6 larval specimens of each instar and a total of 36 specimens were measured. In addition, the same structure was measured three times in each larval specimen. The sizes of the structures were measured using Image J 1.53. The mean and standard error were calculated using SPSS 26.0. For the eggs, we adopted the nomenclature used by Vargas [21] and Hu [22]. The head chaetotaxy was based on the work of Rougerie [23], while the antennae and mouthparts were categorized according to guidelines provided by Liu [24]. Ocelli were labeled according to Wang [25], and thoracic legs were labeled as per Matraj [26]. The abdominal and anal prolegs were classified in accordance with Hasenfuss [27]. To describe the setae shapes, we adhered to the findings of Wang [28], and the pupal morphology was described as recommended by Goel [29].

## 3. Results

### 3.1. Morphology of Eggs

**Figure 1:**
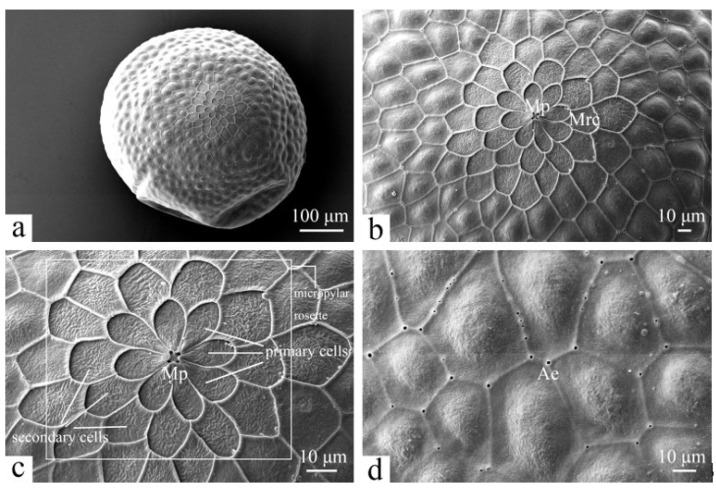
Scanning electron micrographs of eggs of *H. cunea*. (**a**) Egg in upper-lateral view; (**b**) upper region of egg enlarged; (**c**) micropylar rosette in upper view; and (**d**) aeropyles on eggshell. Ae—aeropyle; Mp—micropyle; Mrc—micropylar rosette cell; white rectangle indicates the micropylar rosette.

The eggs have a mean diameter of 0.50 ± 0.08 mm, are spherical in shape, pale yellow to yellowish green, and have a rough surface (Figure 1a). The eggshell features a dense network of continuous, irregular polygonal cells, with four micropyle openings located within the upper region (Figure 1b). The polygonal cells can be distinguished by their thin but clearly defined boundaries. The micropylar rosette consists of two circles of cells, comprising eight primary cells and approximately twelve secondary cells, surrounding the micropyles (Mp) (Figure 1c). In regions outside the micropylar rosette, the cells on the surface of the eggshell are slightly elevated (Figure 1d). Aeropyles (Ae) are distinctly visible at the junctions of the boundaries of the polygonal cells on the surface of the eggshell (Figure 1d). Notably, the aeropyles are larger and more pronounced at the corners of the polygonal cells than those at the edges (Figure 1d).

### 3.2. Morphology of Larvae

#### 3.2.1. Antennae

**Figure 2:**
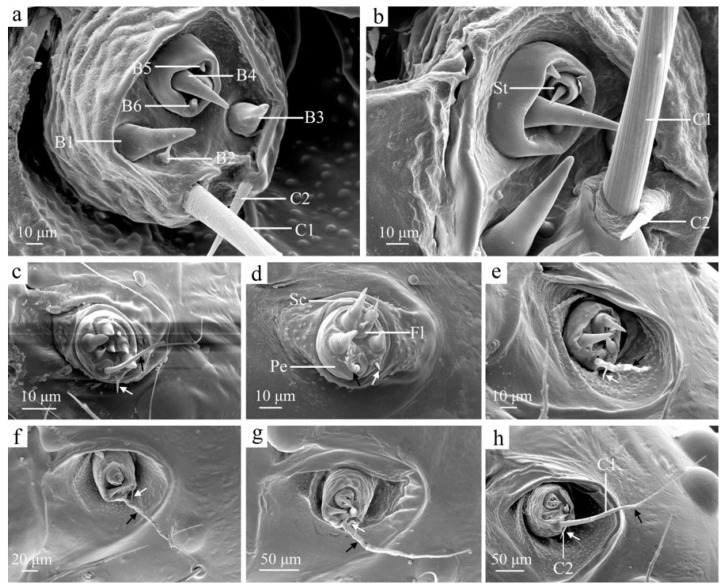
The antennae of the larvae of *H. cunea*. (**a**) Sixth-instar antennae, enlarged; (**b**) sixth-instar antennae, enlarged; (**c**) first instar; (**d**) second instar; (**e**) third instar; (**f**) fourth instar; (**g**) fifth instar; (**h**) sixth instar. B1–6—sensilla basiconica 1–6; C1–2—sensilla chaetica 1–2 on pedicel; Fl—flagellum; Pe—pedicel; Sc—scape; St—sensilla styloconica on flagellum; black arrows indicate changes in C1; white arrows indicate changes in C2.

The antennae of *H. cunea* larvae are located on the lateral sides of the mandibles and are divided into three segments (Figure 2d). The first segment, known as the scape (Sc), is very short and is positioned at the base. The second segment, the pedicel (Pe), contains three sensilla basiconica (B1, 45.13 ± 2.76 μm long and 18.66 ± 1.02 μm wide; B2, 14.71 ± 1.74 μm long and 7.76 ± 0.73 μm wide; and B3) and two sensilla chaetica, C1 and C2 (Figure 2a,h). C2 is 43.97 ± 1.89 μm long and 6.90 ± 0.42 μm wide. It is situated near the apical margin of the pedicel. C1 is 347.96 ± 11.01 μm long and 12.19 ± 0.57 μm wide. It is thicker, longer, and more finely striated than C2, featuring a central spiral and microspines. In contrast, C2 tapers from base to tip and has a smooth surface. B2 is smaller and thicker at the base, tapering sharply toward the tip. B1 and B3 are thicker, with blunter tips and smooth surfaces, and positioned on either side of the pedicel. The third segment, the flagellum (Fl), is very small and located at the tip of the pedicel. Unlike the pedicel, the flagellum contains four sensilla basiconica (B4, 34.96 ± 2.13 μm long and 11.73 ± 0.75 μm wide; B5, 5.88 ± 0.31 μm long and 4.18 ± 0.15 μm wide; and B6), as well as a sensilla styloconica (St, 12.36 ± 0.82 μm long). St is cylindrical at the base, concave in the center, and tapers to a blunt tip with a smooth cuticle (Figure 2b). B4 has a smooth surface with a thin, pointed tip, while B5 is much smaller than B4, featuring a rounded and blunt top. B6 is slightly larger than B5 and is shaped similarly to B2 (Figure 2a,b).

As the larvae mature, the overall size of the antennae increases. The sensilla chaetica C1 and C2 exhibit significant growth in both their length and base width (Figure 2, Table 1). No other noticeable changes in the antennae occur with age.

#### 3.2.2. Ocelli

**Figure 3:**
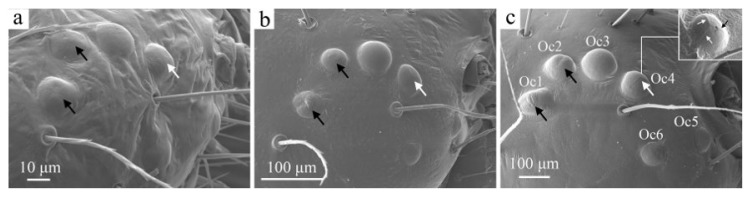
The ocelli of the larva of *H. cunea* in lateral view. (**a**) First instar; (**b**) fourth instar; (**c**) sixth instar. Oc1–6, ocellus 1–6; large black arrows indicate changes in Oc1 and Oc2; large white arrows indicate changes in Oc4; little black and white arrows indicate the locations of the ridges of Oc4.

The larvae possess six pairs of ocelli (Oc1–Oc6, Figure 3c), which are generally of equal size. Ocellus Oc1 is located at the outermost part of the head, while ocelli Oc1–Oc4 are arranged sequentially in a curved pattern. Ocellus Oc6 is positioned below Oc4; Oc5 is located anteriorly near the base of the antennae. The distance between Oc1 and Oc2 is approximately 1.5 times greater than that between Oc2 and Oc3, while Oc4 and Oc6 are equidistant from Oc5 and Oc6. The surface of Oc4 is divided into three sections marked by slightly prominent ridges that radiate from the center. The surfaces of Oc1 and Oc2 are slightly concave (Figure 3c).

As the larvae age, the ocelli become increasingly prominent and distinct, although their number and positioning remain consistent across all instars (Figure 3). The ridges of the ocelli begin to develop in the fourth instar, although they are not very pronounced until the sixth instar. Changes in the morphology of Oc1 and Oc2 are similar to those observed in Oc4.

#### 3.2.3. Mouthparts

**Figure 4:**
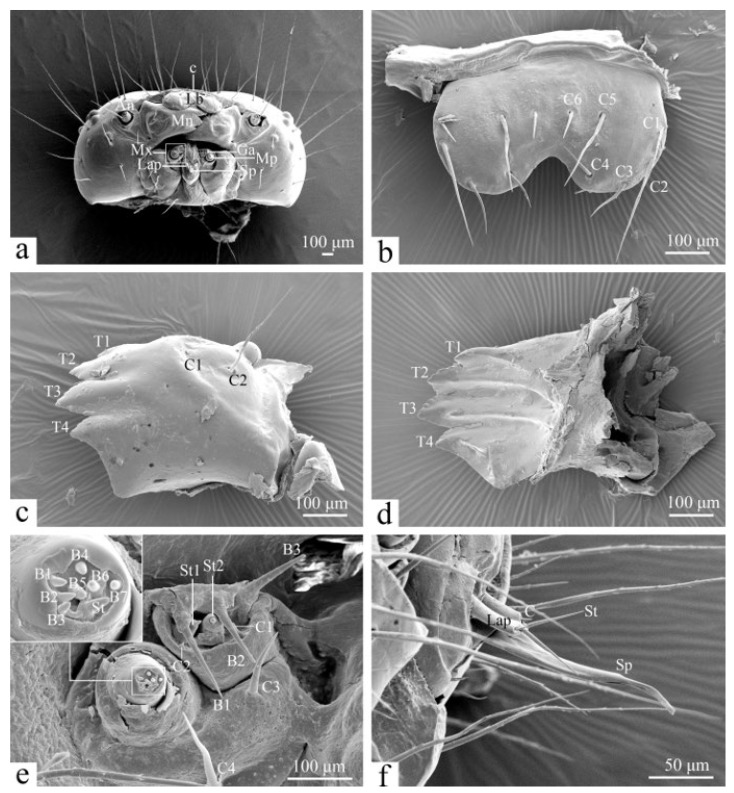
The mouthpart of a sixth-instar larva of *H. cunea*. (**a**) Head in frontal view; (**b**) labrum in dorsal view; (**c**) left mandible in outer view; (**d**) right mandible in inner view; (**e**) maxilla in frontal view; (**f**) labium in lateral view. B1–7—sensilla basiconica 1–7 on maxilla or galea; c—clypeus on head; C—sensilla chaetica on labial palp; C1–6—sensilla chaetica 1–6 on mandible—maxilla or labrum; Ga—galea; Lb—labrum; Lap—labial palp; Mn—mandible; Mp—maxillary palp; Mx—maxilla; Sp—spinneret; St—sensilla styloconica on maxillary palp or labial palp; St1–2—sensilla styloconica 1–2 on galea; T1–4—teeth 1–4.

The mouthparts of *H. cunea* larvae are hypognathous and consist of a labrum, a pair of mandibles, a pair of maxillae, and a labium (Figure 4a).

The labrum (Lb) is 320.34 ± 5.71 μm long and 538.78 ± 1.70 μm wide. It is “W”-shaped with six pairs of symmetrical sensilla chaetica on its surface, with the clypeus (c) connecting to the labrum. The positions of the six symmetrical pairs of sensilla chaetica (C1–C6) are illustrated in Figure 4b. C1 is 91.60 ± 5.24 μm long, C2 is 259.98 ± 6.68 μm long, and C3 is 122.22 ± 2.64 μm long. C1 and C2 are positioned in close proximity, while C3 is farther away; however, all three are located at the lateral margin of the labrum. C4 is 95.68 ± 1.20 μm long, C5 is 188.50 ± 4.80 μm long, and C6 is 73.55 ± 2.74 μm long. They are situated near the median area of the labrum, with C4 slightly closer to the anterior margin. C2 is the longest, followed by C5. The labrum’s ability to move back and forth aids the feeding process.

The mandibles (Mn) are 555.28 ± 6.14 μm long and 410.39 ± 5.59 μm wide. They are inferior to the labrum, and possess four distinct serrated teeth (T1–T4) (Figure 4c,d). The base of each mandible is concave at the anterior margin and elevated in the middle, featuring two sensilla chaetica: C1 is 135.92 ± 5.50 μm long, and C2 is 260.51 ± 3.24 μm long. C2 is longer than C1 (Figure 4c).

The maxillae (Mx) are situated between the mandibles and the labrum and comprise cardo, stipes, maxillary palps (Mp), galea (Ga), and lacinia (Figure 4a). The maxillary palps and galea are shorter and cylindrical in shape. The maxillary palps are conical at the tip and bear seven sensilla basiconica (B1–B7) and one sensillum styloconicum (St) at the apical rounded depression (Figure 4e). A sensillum chaeticum (C4, 142.39 ± 1.95 μm long) is located on the cardo, while C3 is found on the stipes and is 109.76 ± 1.26 μm long. The galea has seven sensilla on its distal surface: two chaetica (C1, 29.68 ± 0.66 μm long and C2, 8.88 ± 0.45 μm long), three basiconica (B1, 131.91 ± 2.75 μm long, B2, 106.15 ± 1.34 μm long, B3, 109.54 ± 1.81 μm long), and two styloconica (St1, 46.74 ± 1.11 μm long and St2). The bases of the sensilla styloconica are conical, with slightly narrower ends and papillate projections, while B2 and B3 have blunter tips.

The labium are inferior to the maxillae, which consists of a pair of labial palps (Lap) and a spinneret (Sp) (Figure 4f). The labial palp is 98.01 ± 1.83 μm long and 12.91 ± 0.35 μm wide; the spinneret is 180.67 ± 2.48 μm long and 37.22 ± 1.79 μm wide. The labial palp contains two types of sensilla: a longer styloconicum (St, 56.68 ± 0.74 μm long) and a shorter chaeticum (C, 11.57 ± 0.34 μm long). The spinneret is long and tubular, with an opening at its tip.

**Figure 5:**
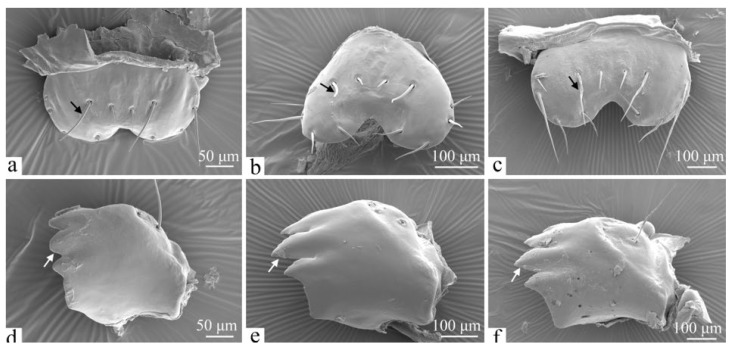
The labrum and left mandible of the fourth- to sixth-instar larvae of *H. cunea*. (**a**) Fourth instar, labrum in dorsal view; (**b**) fifth instar, labrum in dorsal view; (**c**) sixth instar, labrum in dorsal view; (**d**) fourth instar, left mandible in outer view; (**e**) fifth instar, left mandible in outer view; (**f**) sixth instar, left mandible in outer view; black arrows indicate changes in sensilla on labrum; white arrows indicate changes in teeth on mandible.

**Figure 6:**
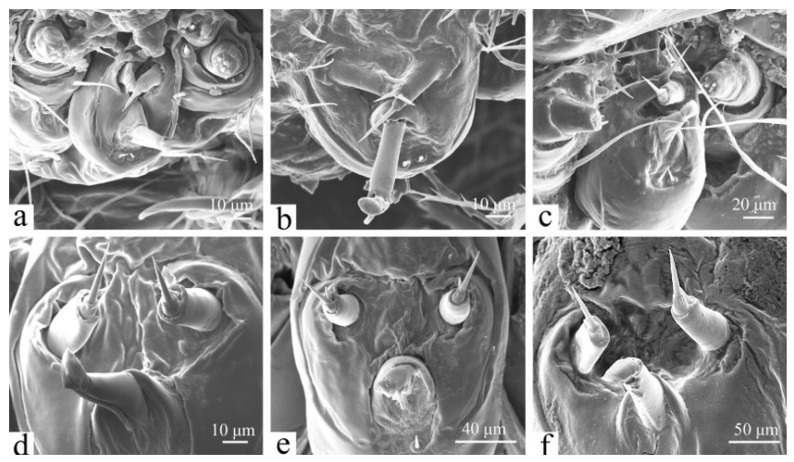
The labium of the larva of *H. cunea* in frontal view. (**a**) First instar; (**b**) second instar; (**c**) third instar; (**d**) fourth instar; (**e**) fifth instar; (**f**) sixth instar.

As the larvae grow, the mouthparts gradually enlarge. The labrum only changes in size (Table 2). There are significant changes in the size of the mandibles (Table 2). In addition to this, the change in the teeth of the mandibles is also very obvious (Figure 5d–f). In the fourth instar, the edges of the teeth are smooth, rounded, and short; by the fifth instar, they begin to sharpen, becoming serrated and remaining so until the sixth instar. The number and positioning of the teeth remain consistent, and there is no significant change in the maxillae except for the enlargement of sensilla. The size of the spinneret increases significantly (Figure 6, Table 3).

#### 3.2.4. Head Chaetotaxy

**Figure 7:**
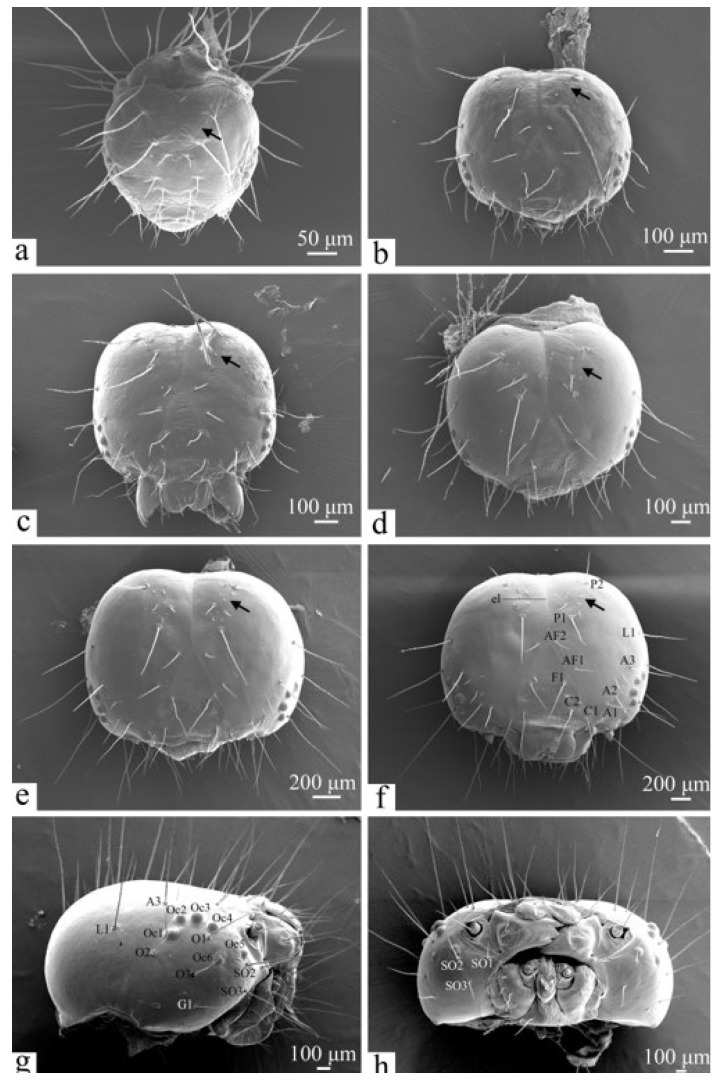
The head chaetotaxy of the larva of *H. cunea*. (**a**) First instar, head in dorsal view; (**b**) second instar, head in dorsal view; (**c**) third instar, head in dorsal view; (**d**) fourth instar, head in dorsal view; (**e**) fifth instar, head in dorsal view; (**f**) sixth instar, head in dorsal view; (**g**) sixth instar, head in lateral view; (**h**) sixth instar, head in frontal view. A1–3—anterior seta 1–3; AF1–2—adfrontal seta 1–2; C1–2—clypeal seta 1–2; F1—frontal seta; G1—genae seta; L1—lateral seta; O1–3—ocellar seta 1–3; Oc1–6—ocellus 1–6; P1–2—posterodorsal seta 1–2; SO1–3—subocellar seta 1–3; black arrows indicate changes in punctures and minute setae.

The larval head is 2062.28 ± 4.44 μm long, 2297.17 ± 8.24 μm wide, and 1255.08 ± 5.41 μm high. It is black, oval-shaped, and relatively smooth, featuring several setae on the cuticle. A shallow inverted “Y”-shaped ecdysial line is present in the center of the head (Figure 7f). Mature larvae exhibit 18 pairs of primary setae on the dorsal surface of the head, arranged bilaterally symmetrically, with the ecdysial line serving as the baseline (Figure 7f). The adfrontal setae group (AF) is located near the frontal area, with AF2 positioned close to the ecdysial line and AF1 situated below AF2. The frontal setae group (F) occupies the middle of the inner triangular area of the frontal angle, containing a single pair of setae (F1). The clypeal setae group (C) is located below group F and above the labrum, consisting of two pairs of setae (C1 and C2), where C1 is close to the intersection of the lateral edge of the labrum with the frontal suture, and C2 is located inside C1. The posterodorsal setae group (P) on the dorsal part of the head features longer setae, with P2 located posterior to P1. Some punctures between P1 and P2 are accompanied by minute setae that are not entirely symmetrical. The anterior setae group (A) is located above the lateral ocelli and consists of three pairs: A1 is above the antennae, A2 is closer to A1, and A3 is further from A2. There are four pairs of asymmetrical minute setae between A3 and A2. The setae near the lateral ocelli form group O, with O1 approximately below Oc4, O2 positioned behind Oc1, and O3 obliquely posterior to Oc6 (Figure 7g). The subocellar setae (SO1, SO2, and SO3) show SO2 immediately adjacent to Oc5, SO1 below the antennae at roughly the same level as SO2, and SO3 below SO1 and SO2, further from the antennae (Figure 7h). The lateral seta (L1) is situated approximately midway between P2 and O2. The lowest setae on the lateral side of the head belong to the genae setae group (G), consisting of a single pair of G1 setae.

As the larvae mature, the head lengthens, broadens, and hardens, and the ecdysial line becomes more pronounced (Figure 7, Table 4). First-instar larvae have long primary setae that are symmetrical on both sides and no secondary setae. In the second instar, secondary setae and punctures begin to emerge, primarily between P1 and P2, though they are asymmetrical and remain indistinct until the third instar. While the number and arrangement of secondary setae vary among specimens, the number and positioning of primary setae remain consistent.

#### 3.2.5. Thoracic Legs

**Figure 8:**
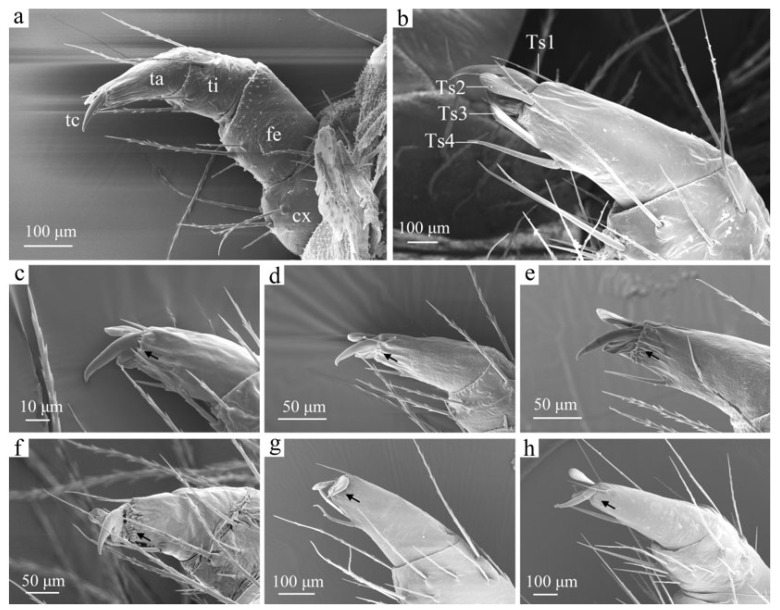
The thoracic legs of a *H. cunea* larva. (**a**) Second instar, thoracic legs in lateral view; (**b**) sixth instar, setae on tarsus; (**c**) first instar, tarsus in lateral view; (**d**) second instar, tarsus in lateral view; (**e**) third instar, tarsus in lateral view; (**f**) fourth instar, tarsus in lateral view; (**g**) fifth instar, tarsus in lateral view; (**h**) sixth instar, tarsus in lateral view. cx—coxa; fe—femur; ta—tarsus; tc—tarsus claw; ti—tibia; Ts 1–4—tarsi seta 1–4; black arrows indicate changes in microspine density.

The larvae possess three pairs of thoracic legs, each of which is divided into five segments: a coxa (cx) is 150.58 ± 1.27 μm long, a femur (fe) is 176.87 ± 1.49 μm long, a tibia (ti) is 130.46 ± 3.85 μm long, a tarsus (ta) is 181.63 ± 2.44 μm long, and there is a tarsal claw (tc) (Figure 8a). The coxa is adorned with numerous setae on both the lateral and mesal surfaces. The anterior surface of the femur also bears setae, while the lateral, posterior, and anterior surfaces of the tibia are similarly equipped. The surfaces of the coxa, femur, and tibia feature a small number of microspines. The tarsus features four setae (Ts1–Ts4) and terminates in a curved, pointed tarsal claw (Figure 8b). Ts4 is longer and thicker than Ts1, while Ts2 is scale-shaped, with longitudinal ridges on its surface, sometimes with serrated edges. Ts3 is rod-shaped and thicker than the others.

As the larvae mature, their thoracic legs grow larger (Figure 8). First-instar larvae possess only a few primary setae on their thoracic legs; however, in subsequent instars, the setae increase in both number and length. The density of microspines at the front end of the tarsus also increases as they evolve into projections. Aside from these changes, no significant differences in thoracic leg morphology are observed among the various larval stages.

#### 3.2.6. Abdominal and Anal Prolegs

**Figure 9:**
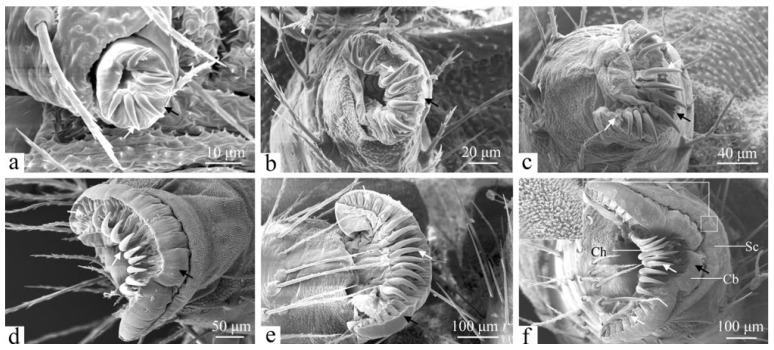
The abdominal prolegs of the larva of *H. cunea* in ventral view. (**a**) First instar; (**b**) second instar; (**c**) third instar; (**d**) fourth instar; (**e**) fifth instar; (**f**) sixth instar. Cb—coronal blisters; Ch—crochets; Sc—subcorona; black arrows indicate changes in undersides of abdominal prolegs; white arrows indicate changes in crochets.

**Figure 10:**
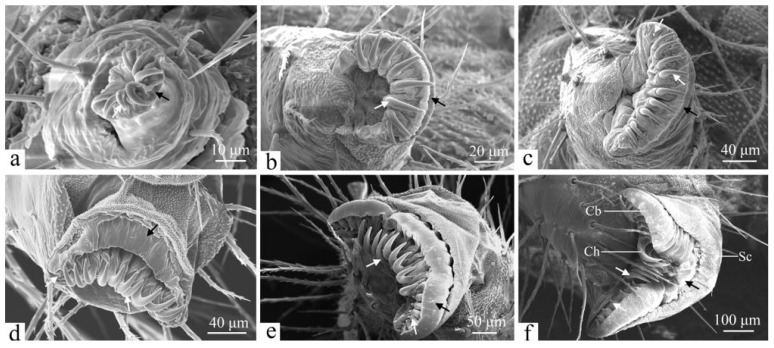
The anal prolegs of the larva of *H. cunea* in ventral view. (**a**) First instar; (**b**) second instar; (**c**) third instar; (**d**) fourth instar; (**e**) fifth instar; and (**f**) sixth instar. Cb—coronal blisters; Ch—crochets; Sc—subcorona; black arrows denote changes in the undersides of anal prolegs; white arrows indicate changes in crochets.

The abdominal and anal prolegs are unsegmented, consisting of a proximal base and a distal base. The lateral and mesal surfaces of the proximal base are densely covered with setae. The underside of the abdominal prolegs features crochets (Ch), coronal blisters (Cb), and a subcorona (Sc) (Figure 9f). The crochet arrangement is characterized by a uniordinal heteroideus midband, with each crochet exhibiting a uniformly patterned surface. The subcorona contains a high density of microtrichia. Structurally, the abdominal and anal prolegs are similar, with a differing number of crochets (Figure 10f). First-instar larvae have 5 to 7 crochets (Figure 9 and Figure 10a), second-instar larvae possess 8 to 10 (Figure 9 and Figure 10b), and third-instar larvae have approximately 12 to 15 (Figure 9 and Figure 10c). For fourth-instar larvae, this number increases to about 16 to 20 crochets (Figure 9 and Figure 10d), while the fifth instar possesses 21 to 23 crochets (Figure 9 and Figure 10e). In the sixth instar, the number of crochets on the prolegs ranges from 25 to 29 (Figure 9 and Figure 10f).

The morphology of the abdominal and anal prolegs varies significantly among different larval instars. In first-instar larvae, the undersides appear relatively simple and smooth, with a limited distribution of crochets (Figure 9 and Figure 10a). In the second instar, coronal blisters and the subcorona begin to emerge (Figure 9 and Figure 10b). By the third instar, small crochets have developed on the lateral sides adjacent to the primary crochets; this trend continues through the sixth instar, with a gradual increase in crochet numbers (Figure 9 and Figure 10c–f). In addition to this, the length of crochets grows gradually (Figure 9 and Figure 10).

#### 3.2.7. Verrucae

**Figure 11:**
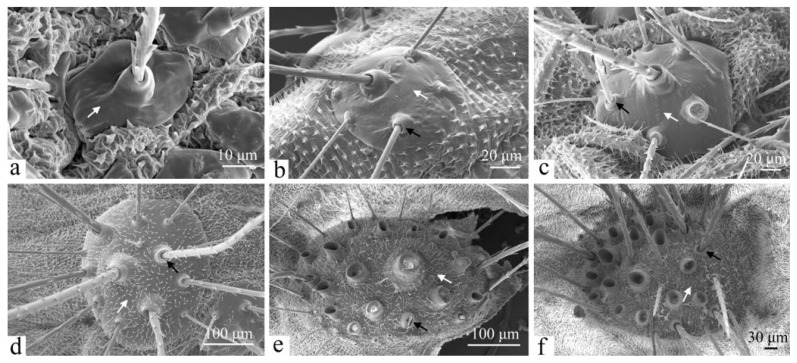
The verrucae of the larva of *H. cunea*. (**a**) First instar; (**b**) second instar; (**c**) third instar; (**d**) fourth instar; (**e**) fifth instar; and (**f**) sixth instar; black arrows indicate changes in the surface on verrucae; white arrows indicate changes in secondary setae.

Larvae have black or yellowish verrucae on their bodies, and their positioning corresponds to that of the primary setae. These structures may be flat or prominent, with well-defined surface boundaries. Each verruca is covered with a varying number of fine setae, and the bases of these setae feature a volcanic structure that connects the internal components of the verruca to the external environment (Figure 11).

As the larvae mature, the verrucae undergo significant changes (Figure 11). In first-instar larvae, the verrucae are small, oval, nearly uniform in size, and have smooth surfaces with a single volcano-shaped protuberance serving as a pore, accompanied by one primary seta (Figure 11a). By the second instar, the size, number, and distribution density of the verrucae have increased. Smaller verrucae emerge between the primary ones, maintaining a smooth surface while giving rise to secondary setae. The primary setae are the thickest and longest, while the secondary setae are thinner and shorter (Figure 11b). In third-instar larvae, while the verrucae remain smooth, the setae thicken and lengthen (Figure 11c). By the fourth instar and beyond, the number of verrucae continues to increase, with irregular surfaces distorted by numerous microtrichia of various shapes. Concurrently, the verrucae continue to grow, and the overall number of setae increases (Figure 11d–f).

### 3.3. Morphology of Pupa

The pupa is 12.91 ± 0.20 mm long, 5.22 ± 0.17 mm wide, and 4.06 ± 0.10 mm high. It is fusiform and opaque, with dark brown or dark reddish-brown coloration and a heavily sclerotized surface. The exterior is rough and sparsely covered with tiny setae.

**Figure 12:**
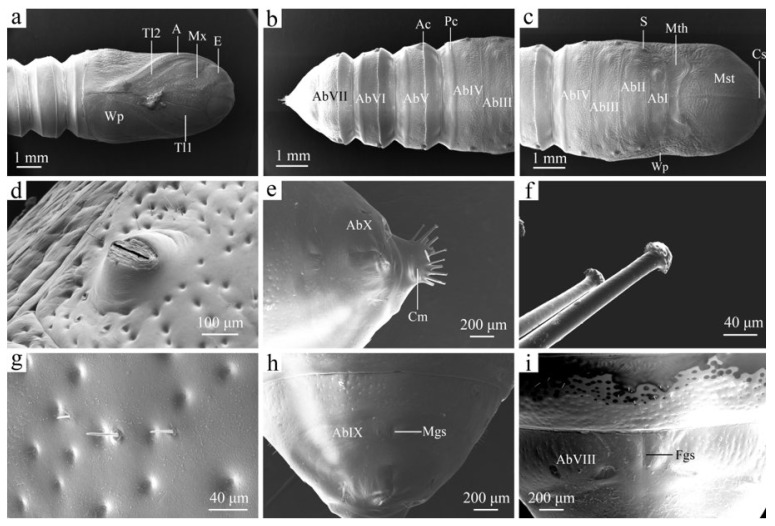
The pupa of the larva of *H. cunea*. (**a**) Pupa in ventral view; (**b**) abdominal segments III–VII in dorsal view; (**c**) pupa in dorsal view; (**d**) spiracle; (**e**) AbX segment in ventral view; (**f**) cremastral seta; (**g**) punctures and fine setae; (**h**) male pupa in ventral view; (**i**) female pupa in ventral view. A—antennae; AbI–AbX—abdominal segments I–X; Ac—anterior crest; Cm—cremaster; Cs—coronal stem; E—eye; Fgs—female genital slit; Mgs—male genital slit; Wp—wing pad; Mth—metathorax; Mst—mesothorax; Mx—maxilla; Pc—posterior crest; S—spiracle; Tl1—prothoracic legs; Tl2—mesothoracic legs.

The coronal stem (Cs) divides the head into two sections. The eyes (E) are positioned at the top of the head; they are oval and slightly convex, and they are surrounded by irregular lines. The maxilla (Mx) is located below the eyes. The antennae (A), which dominate the head and thorax, are located on either side, featuring oval protuberances of varying sizes, and the central portion is adorned with irregular stripes. The tips of the antennae converge at the center of the thorax (Figure 12a,c).

Thoracic segmentation is evident on the dorsal surface of the pupa (Figure 12a,c). On the dorsal side, the prothorax is significantly reduced, while the mesothorax (Mst) and metathorax (Mth) are clearly defined. The coronal stem is extended and prominently visible from above. The wing pads (Wp), originating from the mesothorax, wrap ventrally and diagonally around the lower portion of abdominal segment IV (AbIV). The metathorax extends along the mesothoracic wing pad and eventually blends into the upper part of segment AbIV. Sparse setae are distributed among numerous punctures on the metathorax. Prothoracic legs (Tl1) and mesothoracic legs (Tl2) are enveloped by the antennae.

The abdomen comprises ten segments (AbI–AbX). The connection between abdominal segments I (AbI) through IV (AbIV) differs from that of segments IV to VIII. The four anterior abdominal segments are closely aligned with the metathorax and are fixed, while segments IV to VIII are retractable, with a greater range of motion (Figure 12b,c). Clear dividing lines can be observed between AbI and AbIV. On AbIV to AbVI, the posterior margins of each segment are elevated, forming a posterior crest (Pc). AbV to AbVII exhibit slight elevation at the anterior margins, creating an anterior crest (Ac) (Figure 12b). On AbII to AbVIII, each spiracle (S) appears as a transverse slit located on a raised node, distributed anteriorly on either side of each segment (Figure 12c). There are seven pairs of spiracles located on the dorsolateral side of the pupa (Figure 12d). The AbX segment narrows and tapers to form a cremaster (Cm), which is adorned with 12 setae (Figure 12e). Each seta features a long axis with an umbrella-shaped tip and is densely covered with numerous projections (Figure 12f). Segments AbI to AbIX are characterized by a high density of punctures but contain few setae (Figure 12g). The genital slits are present in segments AbVIII or AbIX. The female’s genital slit is located at the anterior edge of segment AbVIII, while the male’s genital slit is found in the middle of segment AbIX; these genital slits differ in length (Figure 12h,i). The female’s genital slit (Fgs) is approximately twice as long as that of the male.

## 4. Discussion

In this study, we utilized environmental scanning electron microscopy to elucidate the ultramorphology of the eggs, larvae, and pupae of *H. cunea*. The eggshells, larval sensilla, abdominal and anal prolegs, crochets, and mouthparts have highly distinctive structures which can serve as a basis for larval identification. Notably, this study attempts to document the ultrastructural changes in *H. cunea* larvae at different instars.

The eggshells of *H. cunea* exhibit many morphological similarities to those of other studied Lepidoptera species [30]. However, the shape and structure of the micropylar region differ from those of other species. In the present study, the micropylar rosette of *H. cunea* eggshells consists of eight primary petaloid cells and has four micropyle openings. Previous studies have shown that the micropylar rosette of *Andala unifascia* (Walker) (Erebidae: Arctiinae) consists of nine primary cells surrounded by secondary cells, featuring five micropyle openings [31]. In addition, the micropylar rosette of *Cladarctia quadriramosa* (Kollar) (Erebidae: Arctiinae) is composed of nine to fifteen primary cells and has five micropyle openings [32]. Thus, the examined species of Lepidoptera display a wide variety of microstructures on the surface of their eggshells, and these characteristics could maybe be used to classify and identify the eggs of Lepidoptera.

Lepidopteran larvae select host plants mainly through head receptors and then complete their feeding activities, and the study of larval head receptors can help in understanding the mechanism of insect feeding. Lepidoptera larval antennae are olfactory and tactile organs, in addition to being able to sense changes in external temperature [33,34]. On the antennae of *H. cunea* larvae, the scape is devoid of sensors, while the pedicel contains two sensilla chaetica and three sensilla basiconica. The structure, type, and distribution of these sensilla are similar to those of other species of Arctiinae, such as *Spilarctia obliqua* (Walker) (Erebidae: Arctiinae) [35]. However, in a study on the larvae of *Drgyia antiqua* (Linnaeus) (Erebidae: Lymantriinae), the pedicel of the antennae has sensilla chaetica and sensilla basiconica, whereas the flagellum has no sensilla distribution, which is different from the results of the present study [36]. This suggests that the distribution of antennal sensilla may be different in larvae of different subfamilies of Lepidoptera.

The structure and function of sensilla chaetica, sensilla basiconica, and sensilla styloconica have been well studied in recent decades. It is reported that the sensilla chaetica located on the larval antennae are suitable for receiving tactile stimuli and are therefore considered mechanoreceptors [37]. The sensilla basiconica on the antennae are thought to have an olfactory function and play an important role in detecting and discriminating between different plant volatiles, thus helping the larvae to localize and select suitable host plants [38]. Based on its function, we hypothesize that the sensilla basiconica can help *H. cunea* larvae find and locate host plants and sense pheromone signaling molecules in the environment [39]. Sensilla styloconica have sensory organs related to food recognition and may be sensitive to temperature and humidity [40].

The mouthparts are feeding organs, and the sensors on the mouthparts help the insect to choose the most palatable foods. *H. cunea* larvae possess six pairs of sensilla chaetica on the labrum, similar to other Lepidoptera. Studies have indicated that these sensors have mechanosensory functions and are particularly sensitive to vibrations and air currents [41]. The mandibles are mechanosensory organs and are essential for larval food handling [42]. The sensilla chaetica on the mandible can help to check for potential food substances when the larva is feeding and may have a defensive function [43]. The teeth on mandibles vary significantly across species of Lepidoptera [44]. *H. cunea* larvae have four pairs of teeth, whereas *Spilarctia casigneta* (Kollar) (Erebidae: Arctiinae) larvae have three teeth [45].

*H. cunea* larvae have three types of sensilla on their maxillae: sensilla chaetica, sensilla basiconica, and sensilla styloconica. The galea of *H. cunea* larvae have two sensilla chaetica, three sensilla basiconica, and two sensilla styloconica, the number and type of which are the same as those of *Lymantria dispar* (Linnaeus) (Erebidae: Lymantriinae) [46]. The sensilla styloconica located on the galea serve as taste receptors, exhibiting minimal variation between species and playing a vital role in the chemoreception associated with feeding in lepidopteran larvae [47]. Additionally, *H. cunea* larvae have seven sensilla basiconica and one sensillum styloconicum on their maxillary palps; these sensors are involved in larval feeding activities. The sensilla basiconica on maxillary palps have been shown to have gustatory function, perceiving plant stimuli or deterrents [48]; the sensilla styloconica on maxillary palps also have taste function, which can identify and select food [49].

The *H. cunea* larvae labium has a pair of labial palps and a spinneret in all instars, with its length increasing with age of instar. The labial palps are mainly used to help the larvae sense food and to aid feeding. It bears a pair of sensilla chaetica and sensilla styloconia, which remain consistent in all instars. In previous studies, these sensors have been suggested to function as taste sensors, which may be related to food selection and feeding in larvae, while others have suggested that they are a class of mechanosensors that sense the hardness of food [50]. *H. cunea* larvae have a longer spinneret, unlike other lepidopteran larvae, which may be related to their own spathe-webbing habits.

The arrangement of setae on the body of Lepidoptera larvae is an important taxonomic feature [51]. The head chaetotaxy of *H. cunea* larvae closely resembles that of other Lepidoptera larvae. In *H. cunea* larvae, the setae are distributed symmetrically relative to the ecdysial line, which is consistent with observations in other lepidopteran larvae, such as *Dione glycera* [21] and *Bunaeopsis licharbas* [23]. This arrangement aligns with the findings reported by Hinton et al. [52].

Crochets are an important taxonomic feature of lepidopteran larvae. In Lepidoptera, the arrangement of crochets on the larval abdominal prolegs are key characteristics used for larval identification. In the present study, *H. cunea* larvae were found to have a uniordinal heteroideus midband of crochets on their abdominal and anal prolegs. The same results were found in previous studies on *Termessa shepherdi* (Newman) (Erebidae: Arctiinae) and *Scolecocampa medara* (Schaus) (Lepidoptera: Erebidae) [53,54]. In addition to this, the number of crochets of *H. cunea* larvae gradually increases with age, with about 25–29 crochets in the sixth instar. This result differs from other species of Erebidae families—for example, *Termessa shepherdi* (Newman) larvae with 30–32 crochets in the sixth instar [53]; *Scolecocampa medara* (Schaus) with 41–45 crochets [54]; and *Hypena opulenta* (Christoph) (Lepidoptera: Erebidae) with 22–30 crochets [55]. This suggests that the number of crochets of sixth-instar larvae may be helpful to identify the species of Erebidae families. The number of crochets in the larvae of *H. cunea* varies at each instar and gradually increases, which may provide an idea for determining larval instars of *H. cunea*.

## 5. Conclusions

In this study, we employed environmental scanning electron microscopy to characterize the ultramorphology of the eggs, larvae, and pupae of *H. cunea*. The ultrastructure of the eggs and pupae is similar to that of other Lepidoptera, but several larval features differ considerably from those found in other Lepidoptera.

The most notable differences between the larvae of *H. cunea* and those of other Lepidoptera are as follows: three types of sensilla are distributed on the larval head—sensilla basiconica, sensilla chaetica, and sensilla styloconica. The mandibles feature four distinct serrated teeth. The number of crochets in each larval instar increases sequentially, with counts ranging from 5 to 7 in the first instar; 8 to 10 in the second, and approximately 12 to 15, 16 to 20, 21 to 23, and 25 to 29 in the third, fourth, fifth, and sixth instars, respectively.

This study attempts to describe the ultrastructural changes in *H. cunea* larvae at different instars. The studied larvae exhibit significant variations in structure, particularly in the abdominal and anal prolegs. The observed changes in the structure and number of crochets provide a preliminary basis for distinguishing between instars. Our results enrich the previous studies on the ultrastructure of *H. cunea* and lay a solid morphological foundation for further exploring the physiological, biochemical, and sensory characteristics and feeding of *H. cunea*, among other mechanisms; they also provide a basis for studying methods for controlling this invasive species.

## Figures and Tables

**Table 1:** The size of C1 and C2 on antennae of *H. cunea* larvae in different instars.

Instar	Sensilla Type	Length (μm)	Width (μm)
1st instar	C1	41.07 ± 0.47	1.95 ± 0.03
	C2	3.95 ± 0.09	1.04 ± 0.04
2nd instar	C1	99.00 ± 1.96	3.25 ± 0.07
	C2	6.77 ± 0.42	1.68 ± 0.05
3rd instar	C1	120.89 ± 2.83	4.31 ± 0.06
	C2	11.50 ± 0.97	2.09 ± 0.05
4th instar	C1	160.30 ± 4.28	5.90 ± 0.10
	C2	24.26 ± 0.79	2.32 ± 0.07
5th instar	C1	196.22 ± 4.25	8.58 ± 0.27
	C2	32.53 ± 1.02	3.28 ± 0.11
6th instar	C1	347.96 ± 11.01	12.19 ± 0.57
	C2	43.97 ± 1.89	6.90 ± 0.42

**Table 2:** The size of labrum and mandible of *H. cunea* larvae in different instars.

Structure	Instar	Length (μm)	Width (μm)
Labrum	4th instar	141.36 ± 0.86	277.46 ± 1.96
	5th instar	257.45 ± 5.92	399.28 ± 7.01
	6th instar	320.34 ± 5.71	538.78 ± 1.70
Mandible	4th instar	252.86 ± 3.46	203.26 ± 3.49
	5th instar	432.57 ± 7.04	314.96 ± 5.95
	6th instar	555.28 ± 6.14	410.39 ± 5.59

**Table 3:** The size of spinneret of *H. cunea* larvae in different instars.

Instar	Length (μm)	Width (μm)
1st instar	16.79 ± 0.39	5.93 ± 0.14
2nd instar	37.09 ± 0.95	8.64 ± 0.14
3rd instar	56.55 ± 1.12	10.07 ± 0.22
4th instar	85.55 ± 1.04	14.20 ± 0.36
5th instar	109.12 ± 2.03	21.92 ± 0.62
6th instar	180.67 ± 2.48	37.22 ± 1.79

**Table 4:** The size of head of *H. cunea* larvae in different instars.

Instar	Length (μm)	Width (μm)	Height (μm)
1st instar	218.06 ± 2.37	250.65 ± 0.67	148.26 ± 0.66
2nd instar	519.78 ± 1.89	586.54 ± 5.36	326.60 ± 0.91
3rd instar	687.62 ± 2.44	796.02 ± 5.08	388.76 ± 3.35
4th instar	910.48 ± 3.93	994.32 ± 2.55	569.28 ± 4.08
5th instar	1364.26 ± 2.56	1552.53 ± 5.00	802.88 ± 4.32
6th instar	2062.28 ± 4.44	2297.17 ± 8.24	1255.08 ± 5.41

## Data Availability

The original contributions presented in the study are included in the article. Further inquiries can be directed to the corresponding author.
